# Anatomy of Mississippi Delta growth and its implications for coastal restoration

**DOI:** 10.1126/sciadv.aar4740

**Published:** 2018-04-11

**Authors:** Elizabeth L. Chamberlain, Torbjörn E. Törnqvist, Zhixiong Shen, Barbara Mauz, Jakob Wallinga

**Affiliations:** 1 Department of Earth and Environmental Sciences, Tulane University, 6823 St. Charles Avenue, New Orleans, LA 70118–5698, USA.; 2 Department of Marine Science, Coastal Carolina University, Conway, SC 29528–6054, USA.; 3 School of Environmental Sciences, University of Liverpool, Liverpool, UK.; 4 Department of Geography and Geology, University of Salzburg, Hellbrunnerstrasse 34, 5020 Salzburg, Austria.; 5 Netherlands Centre for Luminescence dating, Soil Geography and Landscape Group, Wageningen University, Wageningen, Netherlands.

## Abstract

The decline of several of the world’s largest deltas has spurred interest in expensive coastal restoration projects to make these economically and ecologically vital regions more sustainable. The success of these projects depends, in part, on our understanding of how delta plains evolve over time scales longer than the instrumental record. Building on a new set of optically stimulated luminescence ages, we demonstrate that a large portion (~10,000 km^2^) of the late Holocene river–dominated Mississippi Delta grew in a radially symmetric fashion for almost a millennium before abandonment. Sediment was dispersed by deltaic distributaries that formed by means of bifurcations at the coeval shoreline and remained active throughout the life span of this landform. Progradation rates (100 to 150 m/year) were surprisingly constant, producing 6 to 8 km^2^ of new land per year. This shows that robust rates of land building were sustained under preindustrial conditions. However, these rates are several times lower than rates of land loss over the past century, indicating that only a small portion of the Mississippi Delta may be sustainable in a future world with accelerated sea-level rise.

## Teaser

Prehistoric rates of land gain in a large portion of the Mississippi Delta are significantly outpaced by present-day rates of land loss.

## INTRODUCTION

**Fig. 1:**
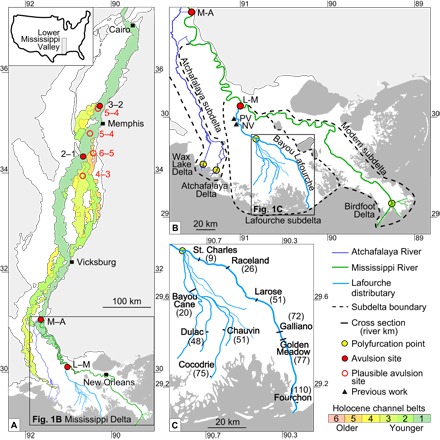
Major past and present paths of the Mississippi River. (**A**) Channel belts and avulsion sites in the Lower Mississippi Valley and Mississippi Delta (for location, see inset), after the study of Saucier (*43*). (**B**) Mississippi Delta, including the Lafourche subdelta, the Modern (Balize) subdelta with the birdfoot delta, and the Atchafalaya subdelta with the Wax Lake and Atchafalaya deltas. Trunk channels that feed these subdeltas branch into multiple distributaries at polyfurcation points, which define the landward limit of bayhead deltas. The two most recent deltaic avulsion sites are the Lafourche-Modern (L-M) and Modern-Atchafalaya (M-A) avulsions. Previous work (*18*) was conducted at Paincourtville (PV) and Napoleonville (NV). (**C**) Location of cross sections, with distance in river kilometers from the Lafourche subdelta polyfurcation point shown in parentheses.

Many of the world’s largest deltas undergo rapid transformations due to reductions in sediment supply (*1*), accelerating rates of sea-level rise (*2*), plus some of the world’s highest subsidence rates (*3*). The Holocene stratigraphic record contains abundant information on the ability of delta plains to grow within the constraints of these controls. However, this archive has only partially been explored, in part due to a historic lack of geochronological tools that are necessary to quantify rates of change. Previous studies have assessed the timing of delta lobe (subdelta) activity through radiocarbon dating of bounding peat (*4*) and shoreline progradation through optically stimulated luminescence (OSL) dating of beach-ridge deposits (*5*). However, delta growth is fundamentally driven by distributary channel activity. Currently available records of delta growth rely largely on instrumental data obtained over the recent decades. For example, the mean land growth rate of the Wax Lake Delta, a recent bayhead delta within the Mississippi Delta, United States (Fig. 1, A and B), has been reported at 0.8 to 3.1 km^2^/year (*6*, *7*). However, the assessment of delta growth over small temporal and spatial scales may reveal little about how river-dominated deltas operate over longer time scales.

Understanding the rates and patterns of delta growth through distributary channel activity is essential for predicting future deltaic land change (*8*), managing sediment resources (*9*), and understanding the effects of human perturbations on deltas (*4*). This information will be of paramount importance in the 21st century as major population and economic centers in large deltas struggle with rapid environmental change. These issues are exemplified well by the Mississippi Delta, where the deposition of clastic sediment by the trunk channel of the Mississippi River (the primary population and infrastructure corridor) is severely hampered by flood protection levees. Despite the growth of new land in the Wax Lake and Atchafalaya deltas and, to a lesser extent, in the birdfoot delta (Fig. 1B), net land loss rates for the delta plain are about 45 km^2^/year, averaged over the past century (*10*). The postindustrial sea-level acceleration is likely a relatively small factor herein. Direct human activities—including reduced sediment delivery, dredging of canals, subsequent saltwater intrusion and wave erosion, and fluid extraction—have played a primary role in the recent degradation of the delta plain (*11*).

Land loss in deltas can be offset by the controlled delivery of new sediment to the delta plain (*9*, *12*, *13*). For example, a $50 billion management plan for coastal Louisiana includes proposals to create new land by the year 2065 through engineered river diversions (*13*) that would reintroduce clastic deposition by means of sediment-laden river water. Developing realistic expectations for the efficacy of these strategies requires an understanding of the natural deltaic processes (for example, distributary channel growth rates and drivers of avulsion) that govern land growth over time scales well beyond decadal-scale instrumental records and the slightly longer historical records (~165 years) (*14*). In addition to information on fluvial sediment loads (*15*) and deltaic sediment retention efficiency (*16*), centennial- to millennial-scale records of rates of land growth in the Mississippi Delta are needed to evaluate whether it is possible to significantly offset the high rates of present-day land loss by means of river diversions. There is currently a lack of field data to answer these questions.

Here, we use OSL dating of mouth bar deposits from the Lafourche subdelta (Fig. 1B) to determine the rates and patterns of growth in the Mississippi Delta. Luminescence techniques enable the direct dating of both subaqueous and subaerial fluviodeltaic deposits (*17*) and have proven successful for dating the deposition of Mississippi Delta sediment (*18*). Mouth bars form as distributaries deliver their sediment load to a receiving basin and reflect deposition of the coarsest sediment fractions as flow decelerates when it meets a standing water body. This results in a sand-dominated deposit that progrades and aggrades to fill the basin (*19*). Vertical accretion of mouth bars occurs more rapidly than can be resolved by OSL. OSL samples taken from any depth within Lafourche subdelta mouth bars therefore reveal the timing of both mouth bar formation and land emergence. Other chronometers, such as radiocarbon dating of peats (*20*), may provide chronologies for the initiation and termination of subdelta activity, but they are less powerful for the direct dating of fluviodeltaic clastic strata. OSL dating of mouth bar sand is therefore the preferred tool to directly capture the time of emergence of new land and thus the progradation of the Lafourche subdelta shoreline.

The Mississippi Delta is composed of a series of subdeltas that formed when quasi-periodic avulsions of major distributaries relocated the depocenter (*21*). The 10,000-km^2^ Lafourche subdelta was active from about 1.6 to 0.6 thousand years (ka) ago (*18*, *20*) under conditions of fairly constant relative sea-level rise. Water and sediment discharge was shared with the Modern (Balize) subdelta after 1.4 to 1.0 ka ago (*22*). The abandonment of the Lafourche subdelta likely preceded the initiation of the Atchafalaya subdelta (*22*, *23*), so river discharge was never shared between these two subdeltas.

We selected the Lafourche subdelta for this study because it is the most recently abandoned subdelta in the Mississippi Delta. The Lafourche subdelta has experienced a complete delta cycle (*24*) and therefore provides an archive for river-dominated delta growth from initiation to termination, yet it has experienced limited reworking compared with older subdeltas. In addition, this system has a well-constrained sea-level history with a long-term sea-level rise trend of 0.6 mm/year (*25*). In the uppermost reach (about 55 river km long), the Lafourche system essentially features one trunk distributary channel that fed sediment to the surrounding delta plain through episodic overbank deposition, including abundant crevassing on top of a widespread wood peat bed (*18*). This demonstrates that the region between the avulsion site (L-M) and the furcation of the trunk channel (Fig. 1B) was subaerial before the initiation of the Lafourche subdelta (*20*). Here, we focus on the lower reach of the subdelta (seaward of the trunk channel) based on 10 cross sections roughly perpendicular to both the main distributary (Bayou Lafourche) and the lesser distributaries (Fig. 1C). Ages are presented in ka relative to 2010.

## RESULTS

### Stratigraphy

**Fig. 2:**
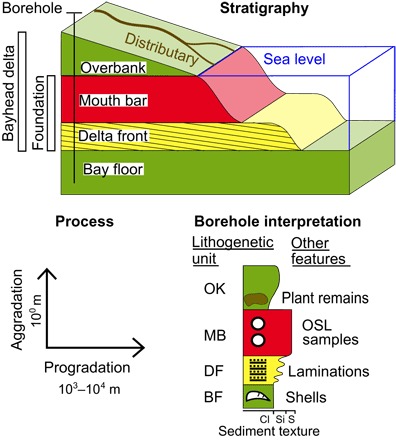
Schematic illustration of the stratigraphy associated with bayhead delta progradation and aggradation. Red, sand (S); yellow, silt (Si); green, clay (Cl); OK, overbank deposits; MB, mouth bar deposits; DF, delta front deposits; BF, bay floor deposits.

The Lafourche trunk channel splits into multiple smaller distributaries at 55 river km downstream of its divergence from the modern Mississippi River. This polyfurcation (that is, a furcation of the distributary network resulting in more than two channels) marks the pre-Lafourche shoreline and produced a distributary network that geomorphologically resembles a bayhead delta. Similar polyfurcations mark the antecedent shorelines of modern bayhead deltas, such as the Wax Lake and Atchafalaya deltas, and give rise to the birdfoot shape of the Modern (Balize) subdelta (Fig. 1B). Downstream of the Lafourche polyfurcation point, the Lafourche distributary system built new land by prograding into a shallow bay (Fig. 2). We refer to the area of new land created during Lafourche activity as the “bayhead delta” (~6000 to 8000 km^2^) and the broader area in which Lafourche sedimentation occurred as the “subdelta” (~10,000 km^2^).

**Fig. 3:**
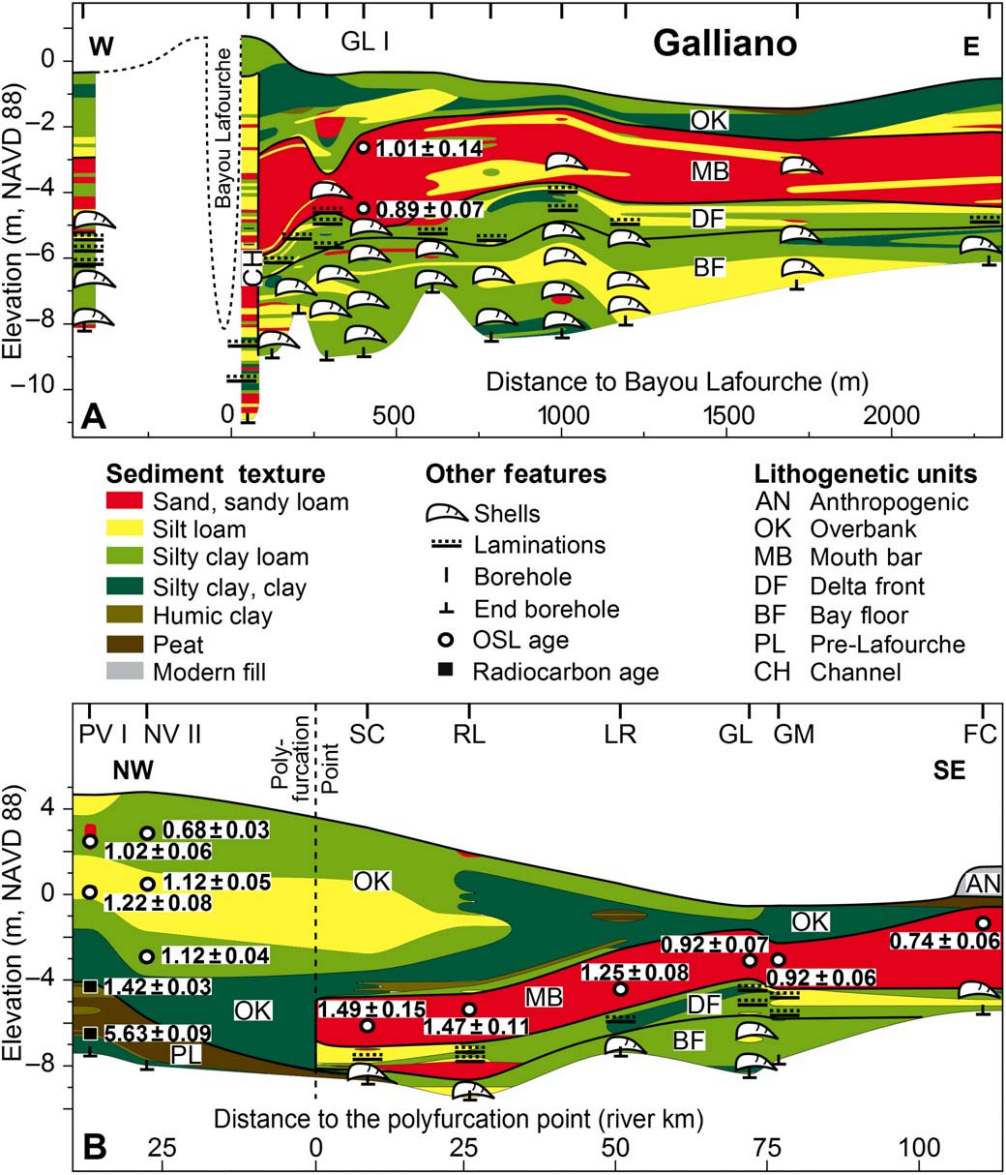
Cross sections illustrating the stratigraphy adjacent to Bayou Lafourche. (**A**) Example of a cross section perpendicular to the main distributary at Galliano; additional cross sections are shown in the Supplementary Materials (fig. S1). Location and orientation of cross sections are shown in Fig. 1 (B and C). (**B**) Cross section parallel to the main distributary of the Lafourche subdelta. Deposits underlying the Lafourche bayhead delta that formed in a subaerial setting are referred to as “Pre-Lafourche.” In (B), weighted mean OSL ages and average sample depths are shown for locations seaward of the polyfurcation point, and the chronology for the PV I and NV II boreholes is from the previous studies (*18*, *20*). Note that the uppermost portion of the overbank unit is highly generalized; for details, see the study of Shen *et al*. (*18*). All ages are presented as thousands of years (ka) ago, relative to 2010.

**Fig. 4:**
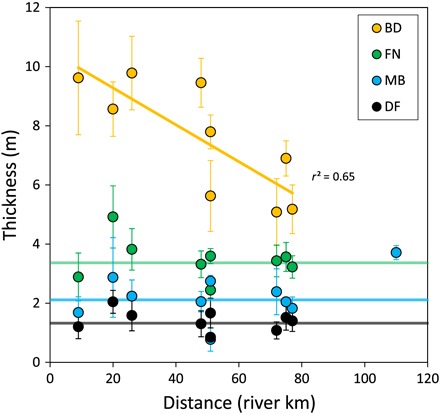
Downstream trend in the thickness of lithogenetic units. The average thickness of the MB and DF deposits for each transect is plotted against distance with reference to the Lafourche polyfurcation point (Fig. 1C). The thickness of the bayhead delta (BD) and foundation (FN) strata is also shown. The colored horizontal lines show the average thickness of the MB, DF, and FN deposits, and the orange line indicates the trend in thickness of the BD deposits. See the Supplementary Materials (fig. S2 and table S2) for uncertainties on the average thickness.

The bayhead delta exhibits a common succession of shell-rich bay-floor muds overlain by 1.3 ± 0.5–m–thick laminated delta front silts and then 2.1 ± 0.8–m–thick mouth bar sands, capped by overbank sediments of varying textures that thin both seaward and away from the channel (Figs. 3 and 4 and fig. S1). Overbank deposits are relatively fine-grained and somewhat organic near the base. In the more mature regions of the subdelta, the overbank unit grades vertically into a patchwork of relatively coarse deposits that pinch out coastward and away from the channel (Fig. 4). This shows that initial, channel-proximal elevation gain in the newly formed bayhead delta was dominated by the deposition of clays, likely through annual flooding. Later, elevation gain was characterized by deposition of predominantly silts associated with crevasse channels. The thickness of bayhead delta strata is similar between the main and lesser distributaries (fig. S2). The combined thickness of mouth bar and delta front deposits (referred to as “foundation deposits”) that aggraded to sea level and subsequently supported the growth of the subaerial delta through overbank deposition is consistent throughout the bayhead delta (Fig. 4).

### Growth patterns

Modern bayhead deltas have been shown to prograde in a radially symmetric pattern at their onset (*26*). This finding is consistent with observational and modeling studies demonstrating that the most seaward portion of a delta is characterized by bifurcations that produce coeval distributaries (*27*, *28*). However, other studies suggest that radial growth of deltas may be restricted to these early stages, whereas more mature systems may prograde in succession by means of repeated avulsions within the subdelta distributary network. Such a mechanism has found support from a widely used Holocene Mississippi Delta radiocarbon chronology (*29*), as well as historical records of the human-modified Po (*30*) and Huanghe (*31*) deltas that feature distributary avulsions within 20 and 100 km of the present-day shoreline, respectively.

**Fig. 5:**
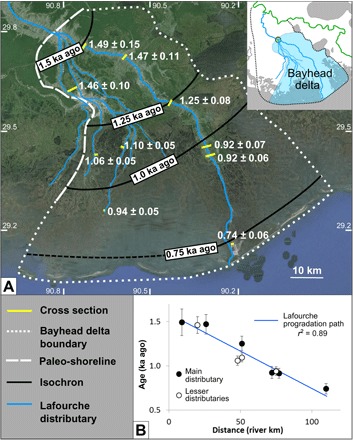
Growth history of the Lafourche bayhead delta. (**A**) Weighted mean OSL ages determine the timing of new land creation through progradation of the bayhead delta of the Lafourche subdelta (inset). The bayhead delta is bounded to the north and west by the paleo-shoreline, to the south by transgressive Lafourche barrier islands, and to the east by open water (interdistributary lakes). (**B**) The progradation history of MB deposits associated with the main channel (filled symbols) and lesser distributaries (open symbols) is fit with a linear regression (*r*^2^ = 0.89).

Our results show that distributary mouth bars of the Lafourche subdelta at similar distances from the polyfurcation point have matching OSL ages (see Materials and Methods), indicating that growth was characterized by coeval distributary channels throughout its period of activity (Fig. 5). Contrary to what has been proposed by the previous work of Frazier (*29*), there is no evidence for avulsions within the distributary network of the Lafourche bayhead delta. We therefore conclude that the Lafourche distributaries formed by means of bifurcation. This demonstrates that radial growth through distributary channel progradation can persist in river-dominated deltas for nearly a millennium. These data also underscore a principle of distributary evolution evident in both modern and past landscapes of the Mississippi Delta: River-dominated delta systems branch at polyfurcation points associated with the paleo-shoreline (Fig. 1B).

### Growth rates

It has been previously hypothesized that progradation slows and ultimately reverses with delta maturity because the area of the delta plain becomes too large to be supported by a constant sediment supply under conditions of constant accommodation creation (*32*). This process of “autoretreat” has been replicated in laboratory and model experiments (*33*) and has been offered as a possible explanation for transgressive successions found in the ancient stratigraphic record (*34*). Autoretreat has been proposed as a fundamental element of any deltaic system where the evolution of the system may be described by the ratio of accommodation creation to sediment supply rates (*35*). Progradation rates of deltas during the late Holocene have been assessed elsewhere (*4*, *36*); however, the autoretreat concept has never been tested in a real-world setting with a well-constrained sea-level history and geochronology.

The Lafourche bayhead delta grew at an average rate of 6 to 8 km^2^/year, associated with distributary mouth bar progradation at a relatively constant rate of 100 to 150 m/year (*r*^2^ = 0.89; see Materials and Methods) throughout most of the Lafourche activity (Fig. 5). This is a surprising result, considering that discharge was shared between the Lafourche distributaries and the modern Mississippi River after 1.4 to 1.0 ka ago (*22*). Furthermore, at least one major crevasse splay in the upstream reach of the Lafourche subdelta extracted a considerable amount of sediment from 0.8 to 0.6 ka ago (*18*). The constant progradation rate of the Lafourche shoreline indicates that autoretreat did not occur in this system during the time period of interest.

### Avulsions

Avulsions constitute the principal mechanism that shift the depocenter within deltas, thereby driving delta evolution over centennial to millennial time scales. Our new results show that avulsions did not occur within the Lafourche subdelta, suggesting that subdeltas function fundamentally differently and should not be seen as miniature versions of the broader delta. Here, we zoom out to the entire Mississippi Delta and Lower Mississippi Valley to identify avulsion sites and to test the degree to which avulsions are preferentially located near a single node (*37*, *38*) versus a broader zone (*39*, *40*), corresponding to the backwater transition where channel-bed deposition is relatively rapid (*39*, *41*).

The link between backwater dynamics, bed aggradation, and avulsion has been described by the backwater number. The backwater number is defined as the backwater length divided by the avulsion length (the channel length between the avulsion site and the shoreline at the time of avulsion) (*42*) and is reported to range from 0.5 to greater than 4 (*39*, *40*). The two most recent avulsion sites within the Mississippi Delta include the partial shift of the modern Mississippi River to the Atchafalaya River (M-A avulsion) and the partial shift of Bayou Lafourche to the modern river (L-M avulsion) (Fig. 1, A and B). The M-A avulsion was initiated at 0.5 to 0.3 ka ago (*22*). The M-A avulsion length is 490 river km (see Materials and Methods), comparable to the backwater length of the modern Mississippi River, yielding a backwater number of roughly 1 (*41*). The L-M avulsion occurred between 1.4 and 1.0 ka ago (*22*). Our data show that the Lafourche bayhead delta had prograded between 20 and 70 km beyond the polyfurcation point at this time, yielding an avulsion length of 75 to 125 km (see Materials and Methods), significantly shorter than the M-A avulsion length. Assuming similar backwater dynamics as in the modern system, the L-M backwater number is roughly 5. The backwater numbers of the two well-constrained avulsions within the Holocene Mississippi Delta are therefore generally compatible with backwater theory, but not with the concept of repeated avulsion around a single, backwater-mediated node.

Evidence of other Holocene Mississippi River avulsions, in the form of relict channel belts, can be found more than 700 linear km inland, within the uppermost reaches of the Lower Mississippi Valley (Fig. 1A) (*43*). Assuming a sinuosity of 1.9 (*44*), this corresponds to avulsion lengths greater than 1300 km. This region has seen considerable (10 m or more) (*43*) Holocene aggradation, making avulsions almost inevitable. The locations of the two most recent avulsion sites in this region are relatively well defined, yet three or more older avulsions likely occurred within an ~250-km linear zone centered around Memphis, TN (see Materials and Methods).

From this evidence, we conclude that avulsions of the Mississippi River are at least partially dictated by fluvial processes that occur far landward of the delta and extend well beyond the backwater transition. Our findings are consistent with observations of avulsion nodes occurring over an ~80-km linear distance and extending beyond the backwater transition in the Rhine-Meuse Delta (*45*), Netherlands, an area with significantly more data to address this problem (*46*). Within the Mississippi Delta, as well as in other muddy, river-dominated deltas, avulsions may be partly steered by factors such as sediment cohesion (*39*), which may drive the river to reoccupy easily erodible (sandy) channel belts (*47*) rather than forging new tracks through cohesive, muddy overbank strata.

## DISCUSSION

The consistent thickness of foundation deposits indicates that the pre-Lafourche bay floor depth was fairly uniform (3.4 ± 0.8 m) and remarkably similar to basin water depths of modern incipient bayhead deltas of the Atchafalaya subdelta (*48*). The Lafourche subdelta is therefore a good analog for present-day processes of bayhead delta growth, such as the proposed river diversions that are planned to convert open water into land. This similarity to the modern system enables a direct evaluation of the ability of present-day depositional systems (that is, incipient bayhead deltas and engineered diversions) to offset contemporary rates of Mississippi Delta land loss. Our finding that distributary networks polyfurcate at the coeval shoreline provides a framework by which the antecedent shoreline and stratigraphy of other river-dominated deltas may be inferred. On the basis of this, we hypothesize that the paleo-shoreline of the Modern (Balize) subdelta may have been positioned near the polyfurcation point of the birdfoot delta (Fig. 1B) at the time of Modern subdelta initiation.

Although our work tests many fundamental principles of delta growth, our results are limited to describing deposits immediately proximal (at most a few kilometers) to distributary channels. The timing of land emergence in the distal, interdistributary flood basins of the Lafourche subdelta was not tested with our approach. It is possible that progradation and land creation rates varied over decadal or even centennial time scales. However, the precision of the OSL ages does not allow for confidently inferring this higher frequency variability. Furthermore, the nature of the discharge split between the Lafourche subdelta and the Modern (Balize) subdelta from 1.4 to 1.0 ka onward is not known.

Despite these limitations, our work makes considerable contributions to the understanding of delta growth, which are relevant to the management of deltas. Avulsions of the Mississippi River are shown to most likely occur over a broad spatial zone that is only partly mediated by backwater dynamics, with a considerable density of avulsion sites 450 to 700 linear km inland that are unrelated to backwater hydraulics. In contrast, because no evidence was found for avulsions in prograding distributary channels, it seems unlikely that new bayhead deltas associated with river diversions will exhibit avulsions. Rather, they can be expected to grow radially by means of bifurcation.

There are a number of potential reasons why autoretreat is not observed in the Lafourche subdelta, including a relatively slow rate of sea-level rise and a relatively high sediment supply, which may reduce the efficacy of autoretreat (*35*). It is also possible that other mechanisms, for example, higher sediment retention efficiency with increasing delta maturity, exert a primary control over delta growth. Alternatively, deltas situated on relatively open coasts and unconstrained by topography may avulse before they enter a state of autogenic decline. Regardless of the mechanism(s) that may enable sustained progradation, our findings raise questions about the applicability of the autoretreat concept to large deltas and their stratigraphic records.

We document high average progradation rates of 100 to 150 m/year and land area creation rates of 6 to 8 km^2^/year within the Lafourche subdelta, sustained for nearly a millennium, that is, rates that are at least two times higher than present-day growth rates in the Wax Lake Delta (*6*). These rates are especially noteworthy considering that the sediment input was shared between the Lafourche subdelta and the Modern (Balize) subdelta (at least during the latter part of its existence). This finding is relevant to coastal planning because it shows that channels with diminished sediment flux, including the proposed river sediment diversions that siphon only a fraction of modern Mississippi River discharge during relatively short time periods, can be very effective in building new land. However, the average prehistoric rates of land growth are several times (by a factor of about 5 to 7) lower than the recent human-enhanced rates of Mississippi Delta land loss (*10*). Although areas beyond the Lafourche subdelta such as the Modern (Balize) subdelta may have also experienced growth during the time period of concern, there was undoubtedly significant decline in other portions of the Mississippi Delta (that is, pre-Lafourche subdeltas); thus, it is unlikely that net growth of the delta plain exceeded 6 to 8 km^2^/year. Furthermore, land building by the Lafourche subdelta occurred under the lowest rates of relative sea-level rise experienced by the Mississippi Delta throughout the Holocene (*25*). Considering recent land loss rates (~45 km^2^/year) (*10*) in combination with the global sea-level rise acceleration (*49*), net land loss in the modern delta will likely continue regardless of coastal restoration strategies, ultimately producing a deltaic landscape that will be very different from the present one.

## MATERIALS AND METHODS

This study used stratigraphic data obtained through hand coring and OSL dating through a combination of well-established and novel methods. Boreholes were drilled using an Edelman hand auger and gouge. Cores were discretized to 10-cm intervals and described in the field with attention to grain size following the U.S. Department of Agriculture texture classification scheme, sedimentary structures, and fossil content, which informed the interpretation of lithogenetic units (see table S1). The surface elevation at borehole sites was obtained from publicly available LiDAR (light detection and ranging) data. OSL samples were captured using a stainless steel Eijkelkamp sampler that prevents light exposure. Below, we describe the OSL dating approach, as well as the calculation of progradation and land change rates, and avulsion lengths.

### OSL sample preparation and measurement

OSL samples were prepared under amber light at Tulane University following standard procedures (*50*, *51*). Luminescence measurements were performed at the University of Liverpool using 1- to 2-mm aliquots of 75 to 125 μm (~110 grains) or 125 to 180 μm (~50 grains) purified quartz sand, adhered to 10-mm stainless steel discs. The coarsest grain-size fraction for which sufficient sediment was available was used. Descriptions of measurement facilities are given in the previously published work (*52*). A standard single-aliquot regenerative-dose (SAR) protocol (*53*, *54*) with a 200° or 220°C preheat, 180°C cut heat, three to four regenerative points, one recuperation point, and recycling checks including infrared (IR) depletion of the OSL signal (table S3) (*55*) was used to extract the equivalent dose (*D*_e_). Note that *D*_e_ herein refers solely to the absorbed radiation dose estimated from luminescence measurement for a single aliquot. Luminescence measurements were made for 40 s over 250 channels. The OSL signal was integrated over the first 0.48 s, and an early background interval, integrated over 0.48 to 1.76 s, was subtracted (*56*). Aliquot acceptance criteria included recycling and OSL IR depletion ratios of 10% (*55*), a maximum test dose error of 20%, and recuperation of 5% relative to the natural signal.

### OSL age calculation

*D*_e_ data sets were cleaned to remove potential outliers before age modeling (see the Supplementary Materials) and then treated with a bootstrap minimum age model (bootMAM) (*57*, *58*) to obtain the paleodose for each sample. The paleodose is defined as the best estimate of the true burial dose (the average dose absorbed by the dated quartz sand grains within the sample since burial). The bootstrap approach provides the benefit of incorporating uncertainty on the width of the *D*_e_ distribution (sigma_b) expected for well-bleached sands in this setting (*57*). To define the sigma_b input to bootMAM, this study used a new method for quantifying overdispersion based on the assumption that at least some samples contain only well-bleached quartz grains. This assumption was supported by initial tests, which showed that some samples (*n* = 5) had overdispersion values equal to or less than those considered characteristic of well-bleached Mississippi Delta sands by previous studies (*18*, *52*). First, each *D*_e_ data set (*n* = 23; see the Supplementary Materials) was analyzed with a central age model (CAM) (*58*), which gives a central value and overdispersion of the *D*_e_ distribution of the sample (table S4). The values for overdispersion obtained through the CAM were grouped by grain size (75 to 125 μm or 125 to 180 μm) and input with their uncertainties into bootMAM (*57*) with sigma_b = [0,0]. The output revealed the overdispersion that is characteristic of the best-bleached samples within a given grain-size fraction. Overdispersion quantified with this approach was 11 ± 3% for 75- to 125-μm sand and 11 ± 4% for 125- to 180-μm sand. The exclusion and addition of samples to the overdispersion analysis are discussed in the Supplementary Materials.

The natural radiation of bulk sediment was determined using activity concentrations of ^40^K and several radionuclides from the uranium and thorium series, measured using a gamma spectrometer at Tulane University (table S5). The dose rate was calculated using standard dose rate conversion (*59*) and cosmogenic contribution (*60*) factors (table S5). No external alpha contribution was included because the outer layer of the quartz grains was removed by etching. Beta dose attenuation was corrected for grain size (*61*), and attenuation due to pore water was calculated (*62*). Water content was measured by drying bulk sediment for each sample in a low-temperature oven, with 5% uncertainty added.

OSL ages were calculated by dividing the paleodose obtained from the bootMAM by the dose rate shown in table S5. Two samples were dated per cross section, and paired ages that agreed within 2σ unshared uncertainty were accepted. One age (St. Charles I-2) was rejected; this is discussed further in the Supplementary Materials. Paired ages and their unshared uncertainties were treated with a weighted mean following the separation of shared (that is, instrument source calibration, dose rate conversion factors, and gamma spectrometer calibration) and unshared (that is, the spread of the *D*_e_ distribution assigned by the age models, dose rate measurement error due to counting statistics, and water content) errors (*63*) to obtain a single age for land emergence at each cross section. Shared errors were returned in quadrature to the uncertainty of the weighted mean ages after application of the weighted mean.

### Progradation and land change rates

The range of the Lafourche bayhead delta progradation rates (100 to 150 m/year) was obtained by dividing the distance between the most landward (St. Charles) and most seaward (Fourchon) cross sections (101 river km) by the minimum and maximum time span between emergence at these localities (0.65 to 0.97 ka, based on the 1σ uncertainty of the weighted mean OSL ages). The land area produced by the Lafourche bayhead delta was obtained by estimating different shoreline positions at the time of Lafourche subdelta abandonment; other boundaries are better constrained (Fig. 5). The minimum area (6000 km^2^) was calculated using the current position of the transgressive barrier island chain. The maximum area (8000 km^2^) was estimated by projecting the Lafourche subdelta beyond the most seaward cross section (Fourchon, 0.74 ± 0.06 ka), assuming a progradation rate of 150 m/year sustained by all distributaries for the final ~150 years of subdelta activity. The contemporary rate of land loss for the deltaic plain was calculated as the sum of areas lost from the Atchafalaya Delta, Barataria, Breton Sound, Mississippi River Delta, Pontchartrain, Teche-Vermilion, and Terrebonne basins over the time period of 1932 to 2016 (*10*).

### Avulsion lengths

Avulsion lengths in the Mississippi Delta are presented in river kilometers, obtained along the center of river channels using Google Earth. The avulsion length range associated with the establishment of the present-day Mississippi River in the Mississippi Delta was obtained from the distance between the L-M avulsion site and the most seaward and landward positions possible for the Lafourche paleo-shoreline at the time of the avulsion (1.4 to 1.0 ka ago) and by placing the timing of Lafourche subdelta initiation at 1.6 ka ago. The most landward position was determined by multiplying the minimum time that the Lafourche subdelta had been active when the L-M avulsion occurred (0.2 ka ago) by the minimum rate of progradation (100 m/year). Multiplying the maximum time (0.6 ka ago) by the maximum rate of progradation (150 m/year) projected the most seaward position of the paleo-shoreline beyond the realistic region constrained by the OSL ages, and so we established this boundary by using the 1-ka isochron (Fig. 5).

Holocene channel belts and their relative chronology have been mapped by Saucier (*43*). Avulsion sites associated with the creation of new channel belts were identified on the basis of the following criteria: (i) likely redirection of all flows to form a new channel belt, rather than partial redirection of flow via bifurcation; and (ii) the most inland departure between two sequential channel belts, rather than a point where channel belts may cross-cut downstream. Distinction was made between avulsion sites that unequivocally met these criteria versus those that were classified as plausible avulsion sites (Fig. 1A). Other avulsions within this region have been suggested by previous work (*47*). However, those phenomena cannot be ruled out as instances of cross-cutting, given the lack of chronologic data. Holocene channel belt avulsion sites were estimated in linear kilometers relative to the modern shoreline using Google Earth and rounded to the nearest 50 km. The sinuosity of the entire Lower Mississippi River is 1.9 (*44*); this value was used to approximate the avulsion lengths as measured along channels.

## Supplementary Material

http://advances.sciencemag.org/cgi/content/full/4/4/eaar4740/DC1

## SUPPLEMENTARY MATERIALS

Supplementary material for this article is available at http://advances.sciencemag.org/cgi/content/full/4/4/eaar4740/DC1 Stratigraphic data for all cross sections Lithogenetic unit thickness calculation OSL dating approach Sample exclusions and additions to analyses Cleaning of outlying aliquots Sample rejection Comparison with previous OSL approach fig. S1. Cross sections illustrating the stratigraphy and OSL ages for all study sites. fig. S2. Thickness of lithogenetic units at main and lesser distributary cross sections. fig. S3. Comparison of mouth bar sand ages estimated using two approaches. table S1. Characterization of lithogenetic units. table S2. Lithogenetic unit thickness. table S3. Details of the SAR protocol. table S4. Overdispersion details, laboratory code, and OSL sample collection year, location, and depth. table S5. Dose rate details and paleodose. table S6. Experimental details of the OSL approach used in the present study versus the approach used by previous studies. table S7. Comparison of OSL ages estimated with two approaches. References (*64*, *65*)
