# Supplementary material for: Anatomy of Mississippi Delta growth and its implications for coastal restoration
Source: Sci Adv. 2018 Apr 11;4(4):eaar4740. doi: 10.1126/sciadv.aar4740 (PMC5895445; doi:10.1126/sciadv.aar4740)
Supplement: http://advances.sciencemag.org/cgi/content/full/4/4/eaar4740/DC1 [file supp_4_4_eaar4740__index.html]

Science Advances | Science Advances

## Supplementary Materials

**This PDF file includes:**

- Stratigraphic data for all cross sections
- Lithogenetic unit thickness calculation
- OSL dating approach
- Sample exclusions and additions to analyses
- Cleaning of outlying aliquots
- Sample rejection
- Comparison with previous OSL approach
- fig. S1. Cross sections illustrating the stratigraphy and OSL ages for all study sites.
- fig. S2. Thickness of lithogenetic units at main and lesser distributary cross sections.
- fig. S3. Comparison of mouth bar sand ages estimated using two approaches.
- table S1. Characterization of lithogenetic units.
- table S2. Lithogenetic unit thickness.
- table S3. Details of the SAR protocol.
- table S4. Overdispersion details, laboratory code, and OSL sample collection year, location, and depth.
- table S5. Dose rate details and paleodose.
- table S6. Experimental details of the OSL approach used in the present study versus the approach used by previous studies.
- table S7. Comparison of OSL ages estimated with two approaches.
- References (*64, 65*)

Download PDF

**Files in this Data Supplement:**

- Adobe PDF - aar4740\_SM.pdf
