# Supplementary material for: Anatomy of Mississippi Delta growth and its implications for coastal restoration
Source: Sci Adv. 2018 Apr 11;4(4):eaar4740. doi: 10.1126/sciadv.aar4740 (PMC5895445; doi:10.1126/sciadv.aar4740)
Supplement: http://advances.sciencemag.org/cgi/content/full/4/4/eaar4740/DC1 [file aar4740_SM.pdf]

Science Advances

AAAS

advances.sciencemag.org/cgi/content/full/4/4/eaar4740/DC1

Supplementary Materials for

Anatomy of Mississippi Delta growth and its implications for coastal restoration

Elizabeth L. Chamberlain, Torbjörn E. Törnqvist, Zhixiong Shen, Barbara Mauz, Jakob Wallinga

Published 11 April 2018, *Sci. Adv.* **4**, eaar4740 (2018)
DOI: 10.1126/sciadv.aar4740

This PDF file includes:

- Stratigraphic data for all cross sections
- Lithogenetic unit thickness calculation
- OSL dating approach
- Sample exclusions and additions to analyses
- Cleaning of outlying aliquots
- Sample rejection
- Comparison with previous OSL approach
- fig. S1. Cross sections illustrating the stratigraphy and OSL ages for all study sites.
- fig. S2. Thickness of lithogenetic units at main and lesser distributary cross sections.
- fig. S3. Comparison of mouth bar sand ages estimated using two approaches.
- table S1. Characterization of lithogenetic units.
- table S2. Lithogenetic unit thickness.
- table S3. Details of the SAR protocol.
- table S4. Overdispersion details, laboratory code, and OSL sample collection year, location, and depth.
- table S5. Dose rate details and paleodose.
- table S6. Experimental details of the OSL approach used in the present study versus the approach used by previous studies.
- table S7. Comparison of OSL ages estimated with two approaches.
- References (*64*, *65*)

Stratigraphic data for all cross sections

Cross sections illustrating the stratigraphy and chronology at all study sites are provided in fig. S1.

Lithogenetic unit thickness calculation

The thickness of lithogenetic units was calculated as the average thickness obtained for all the boreholes in each cross section, based on the upper and lower limits of stratigraphic features that define the overbank, mouth-bar, and delta-front deposits. Lithogenetic unit definitions are provided in table S1. The bayhead-delta deposits include the overbank, mouth-bar, and delta-front deposits. The foundation deposits include the mouth-bar plus delta-front deposits.

The boundaries between lithogenetic units are generally very clear, and boreholes were excluded from the analysis if boundaries were unclear or anomalous (e.g., unconformities due to channel scour). Boreholes from the Bayou Cane (n=1) and the Galliano (n=2) cross sections were excluded from average lithogenetic unit thickness calculations for these reasons. The average thickness and associated standard deviation of lithogenetic units was determined for each cross section (Fig. 4, fig. S2), with 2% uncertainty added for non-vertical drilling. Non-vertical drilling can only increase the observed depth (i.e., it is an asymmetric error). This was addressed by reporting 99% of the measured depth and adding 1% of this depth to the uncertainty. Delta-front, mouth-bar, and foundation deposits of the main and lesser distributary cross sections were found to have comparable thicknesses (fig. S2). Thicknesses of overbank deposits at the Fourchon and Dulac cross sections were corrected to remove the contributions from anthropogenic fill.

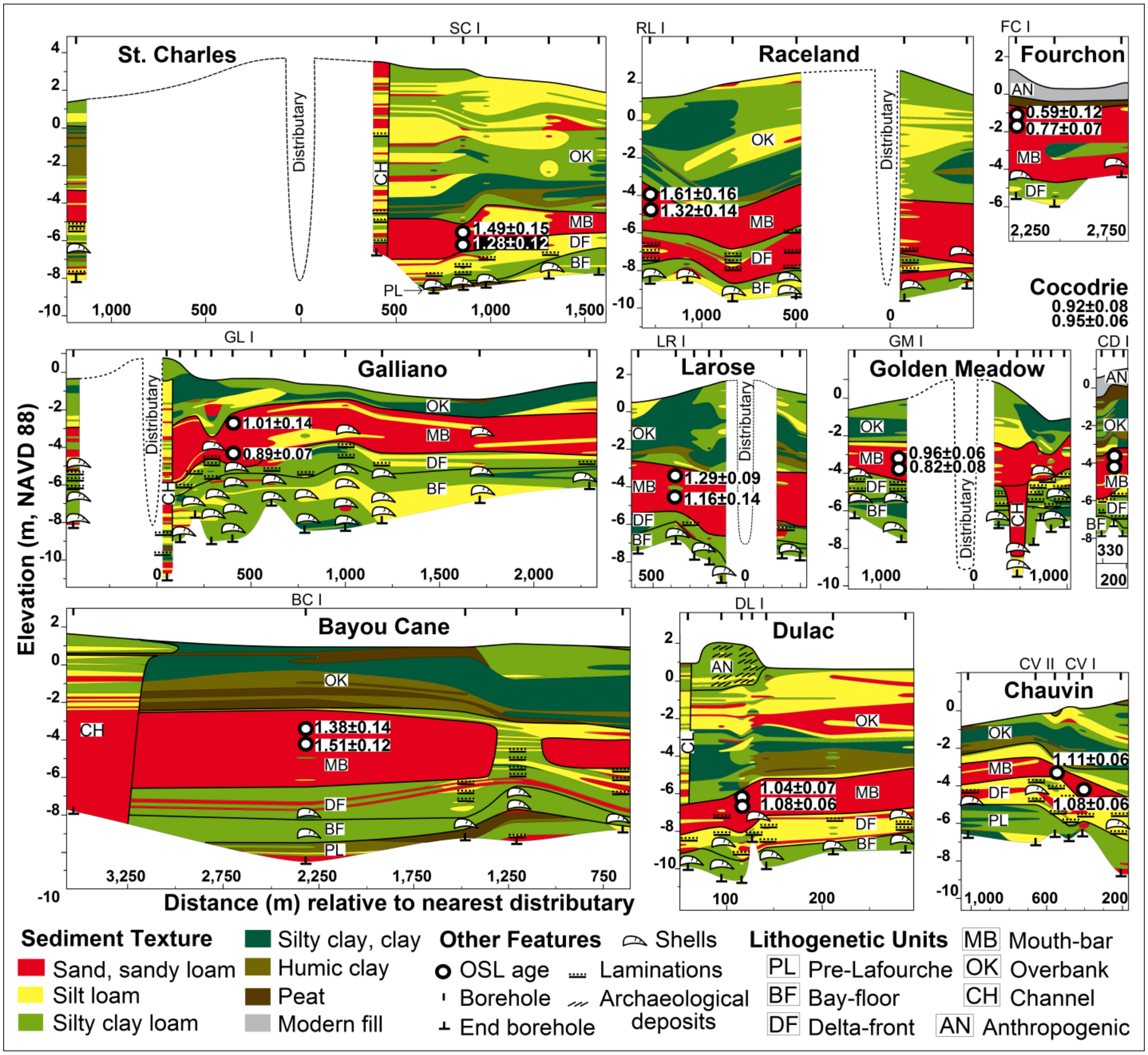

fig. S1. Cross sections illustrating the stratigraphy and OSL ages for all study sites. Location and orientation of cross sections are shown in Fig. 1B; the western side is on the left. Distance (in m) is relative to the nearest distributary channel. Deposits underlying the Lafourche bayhead delta that formed in a subaerial setting are referred to as "Pre-Lafourche". One rejected OSL age is shown in white font on a black background.

table S1. Characterization of lithogenetic units. Lithogenetic units were recognized on the basis of sediment texture, sedimentary structures and other features identified in the cores.

**Lithogenetic unit**	**Dominant sediment texture**	**Sedimentary structures and other features**
Channel deposits	Sand, sandy loam	Sandier toward center of channel belt, clay laminations
Overbank deposits (natural-levee, crevasse-splay, and flood-basin deposits)	Silty clay loam, silt loam, silty clay, clay, humic clay, peat	Highly variable, may contain wood and herbaceous material
Mouth-bar deposits	Sand (very fine to medium)	Homogenous
Delta-front deposits	Clay, silty clay, silty clay loam, silt loam	Laminations, reworked organics, coarsening-upward
Bay-floor deposits	Clay, silty clay, silty clay loam	*Rangia cuneata*, storm-reworked layers of shell hash
Anthropogenic fill	Highly variable	Gravel, shells, unconsolidated dredge spoil, modern garbage (glass, plastic)
Pre-Lafourche subaerial deposits	Humic clay, peat, silty clay, silty clay loam, silt loam, sand	Relatively consolidated clastic or organic deposits, wood and herbaceous material, stratigraphically below delta-front or bay-floor deposits

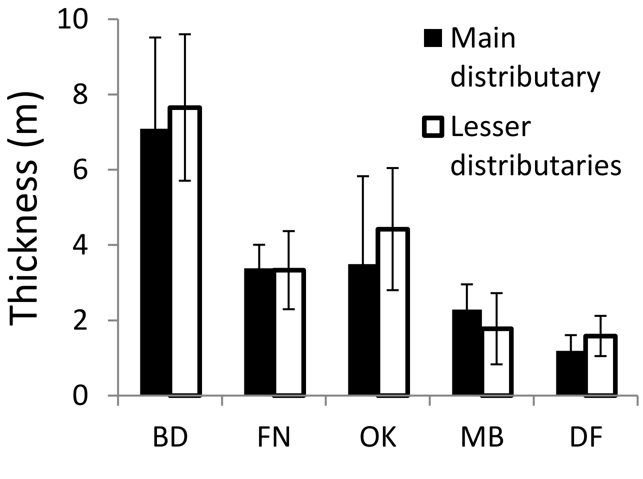

fig. S2. Thickness of lithogenetic units at main and lesser distributary cross sections. Comparison of the thickness of overbank (OK), mouth-bar (MB), and delta-front (DF) as well as the interpreted bayhead-delta (BD) and foundation (FN) deposits. Also see table S2 for more detailed data.

table S2. Lithogenetic unit thickness. Average thickness (uncertainty represents one standard deviation) of the bayhead-delta, foundation, overbank, mouth-bar, and delta-front deposits for each cross section. Distances from the polyfurcation point (PP) are shown in river kilometers.

**Transect**	**Distance from PP (river km)**	**Bore-holes (n)**	**Bayhead-delta deposits (m)**	**Foundation deposits (m)**	**Overbank deposits (m)**	**Mouth-bar deposits (m)**	**Delta-front deposits (m)**
**Main Distributary**	**n.a.**	**42**	**7.1 ± 2.4**	**3.4 ± 0.6**	**3.5 ± 2.3**	**2.3 ± 0.7**	**1.2 ± 0.4**
St. Charles	9	7	9.6 ± 1.9	2.9 ± 0.8	6.7 ± 1.4	1.7 ± 0.5	1.2 ± 0.4
Raceland	26	6	9.8 ± 1.2	3.8 ± 0.7	5.9 ± 0.9	2.2 ± 0.5	1.6 ± 0.5
Larose	51	7	7.8 ± 0.6	3.6 ± 0.3	4.2 ± 0.7	2.7 ± 0.2	0.8 ± 0.1
Galliano	72	11	5.1 ± 1.1	3.4 ± 0.5	1.7 ± 0.8	2.4 ± 0.8	1.1 ± 0.3
Golden Meadow	77	8	5.2 ± 0.8	3.2 ± 0.4	2.1 ± 0.7	1.8 ± 0.4	1.4 ± 0.4
Fourchon	110	3	n.a.	n.a.	0.5 ± 0.2	3.8 ± 0.2	n.a.
**Lesser Distributaries**	**n.a.**	**20**	**7.7 ± 1.9**	**3.3 ± 1.0**	**4.4 ± 1.6**	**1.8 ± 0.9**	**1.6 ± 0.5**
Bayou Cane	20	4	8.6 ± 0.9	4.9 ± 1.1	4.0 ± 0.9	2.9 ± 1.3	2.0 ± 0.4
Dulac	48	7	9.5 ± 0.8	3.3 ± 0.5	6.1 ± 0.7	2.1 ± 0.3	1.3 ± 0.4
Chauvin	51	6	5.6 ± 1.2	2.4 ± 0.3	3.2 ± 1.1	0.8 ± 0.4	1.7 ± 0.5
Cocodrie	75	3	6.9 ± 0.6	3.6 ± 0.5	3.3 ± 0.2	2.0 ± 0.1	1.5 ± 0.4
**All boreholes**	**n.a.**	**62**	**7.3 ± 2.3**	**3.4 ± 0.8**	**3.8 ± 2.2**	**2.1 ± 0.8**	**1.3 ± 0.5**

OSL dating approach

The measurement protocol (table S3), values for overdispersion (table S4) obtained with the central age model (CAM) (*58*), and dose rate details and paleodoses (table S5) are provided here. Details of the OSL analyses, and comparison of ages with those produced using different methods are also discussed. While samples for this project were collected between 2013-2016, OSL ages are reported relative to 2010, to be consistent with previous, closely related work (*18, 22*).

Sample exclusions and additions to analyses

Of the 20 samples measured for this project, one (Galliano I-2) was excluded from the overdispersion analysis (table S4) because of inconsistent mask size. Two grain size fractions of St. Charles I-2 were included in the overdispersion analysis. Three samples of 75-125 µm quartz sand sampled at the Dulac cross section and measured for a separate project (Dulac I-3, Dulac I-4, Dulac I-5) were included in the overdispersion analysis to increase the data available for this grain size fraction.

Cleaning of outlying aliquots

Cleaning of D_e datasets was tested to remove outlying aliquots beyond 4, 3, and 2 standard deviations from the sample mean. Samples Galliano I-1 and Galliano I-2 were excluded from the cleaning analysis because of variable aliquot sizes. We found that a 4 standard deviation cleaning was optimal for treatment of the D_e datasets prior to input into CAM for overdispersion quantification, because this removed 1% of aliquots which were exclusively high-D_e outliers. Prior to input into the bootstrap minimum age model (BootMAM) (*57*,*58*) for paleodose estimation, a 3 standard deviation cleaning was applied. This removed 6% of the total aliquots. Of the aliquots removed by a 3 standard deviation cleaning, 99% were high-D_e aliquots which likely do not represent the depositional age of the sediments, and 1 % were low-D_e aliquots. We found that a 2 standard deviation cleaning removed 24% of aliquots comprised of 82% high-D_e and 18% low-D_e aliquots. This was deemed to be too much lost information.

Sample rejection

The D_es of two grain size fractions (75-125 and 125-180 μm) of St. Charles I-2 were measured because the coarser (125-180 μm) fraction of sediment was found, after measurements, to have too little material to produce a sufficient number of D_es for reliable age model statistics. Both fractions of St. Charles I-2 produced ages that agreed with the age of another sample from the same unit (St. Charles I-1) within 2σ unshared uncertainty. Yet, St. Charles I-2 ages were younger than expected given their stratigraphic context, regardless of the burial dose estimation approach. Therefore, we rejected St. Charles I-2 and used only one sample (St. Charles I-1) to define the timing of land emergence at this cross section.

Comparison with previous OSL approach

This study employed OSL dating methods based on the newest insights (*56*,*57*) with regard to dating young fluvial sediments. As such, the methods used in this study differ from methods used previously to date late Holocene Mississippi Delta deposits (*18*,*22*) in terms of background integration interval, aliquot acceptance criteria, cleaning of aliquots, age model usage, and radionuclide conversion factors applied to dose rate calculations (table S6). To facilitate comparisons of mouth bar sand ages produced by this study with previously published ages for Mississippi Delta deposits, we also analyzed the mouth bar samples using methods applied by previous work (*18*,*22*). Mouth bar sand ages produced with both approaches agreed within 1σ unshared uncertainty (see Materials and Methods), with the exception of Cocodrie I-2 (fig. S3; table S7). Similarly, all weighted mean ages agreed between the two approaches, with the exception of the Cocodrie cross section (river km 75). However, the ages produced with the present approach were systematically lower than those calculated using the previous approach, for the majority (18 out of 21) of the grain size fractions measured for this study. Weighted mean ages produced with the present approach were lower than those produced with the previous approach for all cross sections except for the Fourchon cross section, where the weighted means obtained with both approaches were nearly identical (fig. S3; table S7). The average age offset

between the two approaches was 70 ± 20 years, calculated as the mean and standard error of the previous approach ages minus the present approach ages. This offset is largely related to improved sampling of the fast OSL component through the early background approach, which avoids age overestimation due to incorporation of other, lesser bleached, OSL components (*56*). Ages produced for this study using the present approach were also characterized by larger uncertainties for samples with limited numbers or irregularly dispersed D_es at the aliquot level. The increased uncertainty proved to be a major advantage in calculating ages for each cross section using a weighted mean of paired ages, because it awards greater representation to the high precision ages, which are also the ages most likely to be correct due to robust D_e datasets.

table S3. Details of the SAR protocol.

Step	Treatment	Details
1	Dose	Natural, 3-4 regenerative doses (2.5 to 15 Gy)*, recuperation (0 Gy), recycling (2.5 to 5 Gy)*, recycling (2.5 to 5 Gy)*
2	Preheat	200 or 220 °C for 10 s*
3	OSL	125 °C for 40 s
4	Test dose	4-5 Gy*
5	Cutheat	180 °C for 0 s
6	OSL	125 °C for 40 s
*the protocol evolved during this project, so a range of values is provided		

table S4. Overdispersion details, laboratory code, and OSL sample collection year, location, and depth. Sample name abbreviations as used in Fig. 3 are also provided. LV = University of Liverpool laboratory code.

**Sample name**	**Lab code**	**Sample collection year**	**Coordinates (x, y UTM, NAD 83, 15 N)**	**Sample depth (m)**	**Overdispersion (%)**
**75 - 125 μm**					
Saint Charles (SC) I-2	LV772	2015	717630, 3295150	9.20-9.25	23.0 ± 2.1
Dulac (DL) I-5	LV802	2015	723225, 3257430	4.44-4.57	20.0 ± 2.2
Dulac (DL) I-4	LV803	2015	723225, 3257430	3.26-3.38	17.5 ± 2.2
Golden Meadow (GM) I-2	LV729	2014	763180, 3256650	3.52-3.58	15.5 ± 2.6
Chauvin (CV) I-1	LV778	2015	733170, 3260590	4.40-4.45	10.5 ± 1.2
Dulac (DL) I-3	LV804	2015	723225, 3257430	2.53-2.68	9.6 ± 1.6
Chauvin (CV) II-1	LV780	2015	733030, 3260630	3.17-3.23	9.6 ± 1.1
**125-180 μm**					
Larose (LR) I-2	LV731	2014	749020, 3275160	5.35-5.40	75.1 ± 7.8
St. Charles (SC) I-1	LV771	2015	717630, 3295150	8.90-8.95	54.2 ± 6.9
Larose (LR) I-1	LV730	2014	749020, 3275160	4.20-4.35	53.0 ± 5.3
Bayou Cane (BC) I-2	LV773	2015	714880, 3279540	4.75-4.80	44.2 ± 4.2
Galliano (GL) I-1	LV658	2013	761900, 3261070	3.15-3.20	43.6 ± 7.5
Galliano (GL) I-2	LV657	3012	761900, 3261070	3.80-3.85	excluded
Saint Charles (SC) I-2	LV772	2015	717630, 3295150	9.20-9.25	42.1 ± 6.8
Raceland (RL) I-1	LV724	2013	732550, 3289450	5.30-5.43	40.1 ± 4.4
Raceland (RL) I-2	LV725	2013	732550, 3289450	5.63-5.74	40.0 ± 4.5
Bayou Cane (BC) I-1	LV774	2015	714880, 3279540	4.50-4.55	35.5 ± 3.3
Fourchon (FC) I-1	LV727	2014	772510, 3227620	2.40-2.58	32.1 ± 4.5
Golden Meadow (GM) I-1	LV728	2014	763180, 3256650	3.45-3.55	29.1 ± 4.4
Fourchon (FC) I-2	LV726	2014	772510, 3227620	2.66-2.81	25.9 ± 4.7
Cocodrie (CD) I-2	LV776	2015	727300, 3238520	4.88-4.98	18.1 ± 2.3
Cocodrie (CD) I-1	LV777	2015	727300, 3238520	4.70-4.77	16.7 ± 1.8
Dulac (DL) I-1	LV801	2015	723225, 3257430	8.40-8.48	10.1 ± 1.2
Dulac (DL) I-2	LV800	2015	723225, 3257430	8.63-8.68	7.7 ± 1.3

table S5. Dose rate details and paleodose. Dose rate parameters include water content, grain size, activities of isotopes derived from the uranium (U) and thorium (Th) chain, and from potassium (40K), the internal dose rate of quartz (Q internal), and the cosmogenic dose rate (D cosm.). The calculated dose rate (D) is given here.

**Sample name**	**Water content (%)**	**Grain size (μm)**	**U (Bq/kg)**	**Th (Bq/kg)**	**40K (Bq/kg)**	**Q internal (Gy/ka)**	**D cosm. (Gy/ka)**	**D (Gy/ka)**	**Paleo-dose (Gy)**
Bayou Cane I-1	23 ± 5	125-180	30.14 ± 0.60	27.46 ± 0.38	536.46 ± 13.91	0.03 ± 0.02	0.11 ± 0.01	2.18 ± 0.11	3.00 ± 0.26
Bayou Cane I-2	21 ± 5	125-180	32.28 ± 0.65	29.15 ± 0.43	497.93 ± 13.12	0.03 ± 0.02	0.10 ± 0.01	2.22 ± 0.11	3.34 ± 0.20
Cocodrie I-1	21 ± 5	125-180	28.83 ± 0.58	26.74 ± 0.40	444.58 ± 11.78	0.03 ± 0.02	0.10 ± 0.01	2.00 ± 0.08	1.83 ± 0.14
Cocodrie I-2	24 ± 5	125-180	33.89 ± 0.68	29.75 ± 0.45	502.08 ± 13.27	0.03 ± 0.02	0.10 ± 0.01	2.17 ± 0.10	2.05 ± 0.06
Chauvin I-1	22 ± 5	75-125	47.51 ± 0.91	41.82 ± 0.53	499.35 ± 13.04	0.03 ± 0.02	0.11 ± 0.01	2.59 ± 0.14	2.79 ± 0.06
Chauvin II-1	27 ± 5	75-125	41.12 ± 0.81	36.03 ± 0.51	542.01 ± 14.26	0.03 ± 0.02	0.13 ± 0.01	2.39 ± 0.12	2.64 ± 0.06
Dulac I-1	22 ± 5	125-180	36.78 ± 0.72	31.75 ± 0.45	523.33 ± 13.73	0.03 ± 0.02	0.05 ± 0.01	2.29 ± 0.12	2.37 ± 0.09
Dulac I-2	22 ± 5	125-180	33.60 ± 0.67	29.48 ± 0.42	507.03 ± 13.28	0.03 ± 0.02	0.05 ± 0.01	2.18 ± 0.10	2.34 ± 0.07
Fourchon I-1	20 ± 5	125-180	18.56 ± 0.41	17.01 ± 0.32	471.00 ± 12.49	0.03 ± 0.02	0.15 ± 0.02	1.82 ± 0.07	1.09 ± 0.21
Fourchon I-2	21 ± 5	125-180	17.65 ± 0.40	16.30 ± 0.34	466.52 ± 12.58	0.03 ± 0.02	0.14 ± 0.01	1.77 ± 0.07	1.39 ± 0.10
Galliano I-1	19 ± 5	125-180	23.65 ± 0.57	21.24± 0.54	484.02 ± 13.13	0.03 ± 0.02	0.10 ± 0.01	1.98 ± 0.09	2.00 ± 0.25
Galliano I-2	19 ± 5	125-180	34.02 ± 0.76	34.68 ± 0.84	468.42 ± 12.78	0.03 ± 0.02	0.12 ± 0.01	2.29 ± 0.11	2.06 ± 0.14
Golden Meadow I-1	22 ± 5	125-180	27.37 ± 0.57	25.78 ± 0.41	504.74 ± 13.37	0.03 ± 0.02	0.13 ± 0.01	2.06 ± 0.09	2.01 ± 0.09
Golden Meadow I-2	24 ± 5	75-125	36.24 ± 0.75	31.88 ± 0.52	550.30 ± 14.76	0.03 ± 0.02	0.13± 0.01	2.34 ± 0.12	1.93 ± 0.16
Larose I-1	23 ± 5	125-180	23.76 ± 0.50	22.26 ± 0.38	511.85 ± 13.56	0.03 ± 0.02	0.11 ± 0.01	1.96 ± 0.08	2.55 ± 0.14
Larose I-2	27 ± 5	125-180	36.68 ± 0.77	34.07 ± 0.58	579.71 ± 15.68	0.03 ± 0.02	0.09 ± 0.01	2.29 ± 0.11	2.69 ± 0.30
Raceland I-1	18 ± 5	125-180	18.21 ± 0.40	18.61 ± 0.34	419.67 ± 11.25	0.03 ± 0.02	0.09 ± 0.01	1.72 ± 0.07	2.79 ± 0.25
Raceland I-2	20 ± 5	125-180	32.37 ± 0.65	31.08 ± 0.45	478.01 ± 12.59	0.03 ± 0.02	0.09 ± 0.01	2.17 ± 0.10	2.90 ± 0.27
St. Charles I-1	21 ± 5	125-180	37.89 ± 0.75	38.28 ± 0.56	476.81 ± 12.65	0.03 ± 0.02	0.05 ± 0.01	2.29 ± 0.11	3.42 ± 0.30
St. Charles I-2	23 ± 5	75-125	41.75 ± 0.83	39.06 ± 0.60	540.39 ± 14.40	0.03 ± 0.02	0.05 ± 0.01	2.50 ± 0.14	2.79 ± 0.20
St. Charles I-2	23 ± 5	125-180	41.75 ± 0.83	39.06 ± 0.60	540.39 ± 14.40	0.03 ± 0.02	0.05 ± 0.01	2.46 ± 0.13	3.15 ± 0.22

table S6. Experimental details of the OSL approach used in the present study versus the approach used by previous studies. (*18*,*22*) that applied OSL dating to Holocene Mississippi Delta deposits.

**Aliquot equivalent doses**	**Present approach**	**Previous approach**
Signal (s)	first 0.48	first 0.48
Background (s)	0.48-1.76	32-40
Use recycled points for fitting	no	yes
Force curve through origin	yes	no
Measurement error (%)	1.7	1.7
Monte Carlo uncertainty[a]	yes, x1000	no
Use errors when applying criteria	yes	no
IR depletion test	10%	10%
Maximum test dose error	20%	no
Maximum paleodose error	no	no
Maximum recuperation	5% (% largest R)	5% (% N)
Tn >3sigma above BG	no	no
**Age model usage**		
Cleaning of aliquot datasets	3 standard deviations	applied 'as needed'
Age model	bootMAM	MAM or CAM
Overdispersion input to age model	11 ± 4% (128-180 µm),<br>11 ± 3% (75-125 µm)	10% for all sand grain sizes
Include error on overdispersion	yes	no
**Dose rate calculations**		
Radionuclide conversion factors	Guérin et al. (2011) (*59*)	Adamiec and Aitken (1998) (*64*)
[a]Duller, G.A.T. (2016) (*65*)		

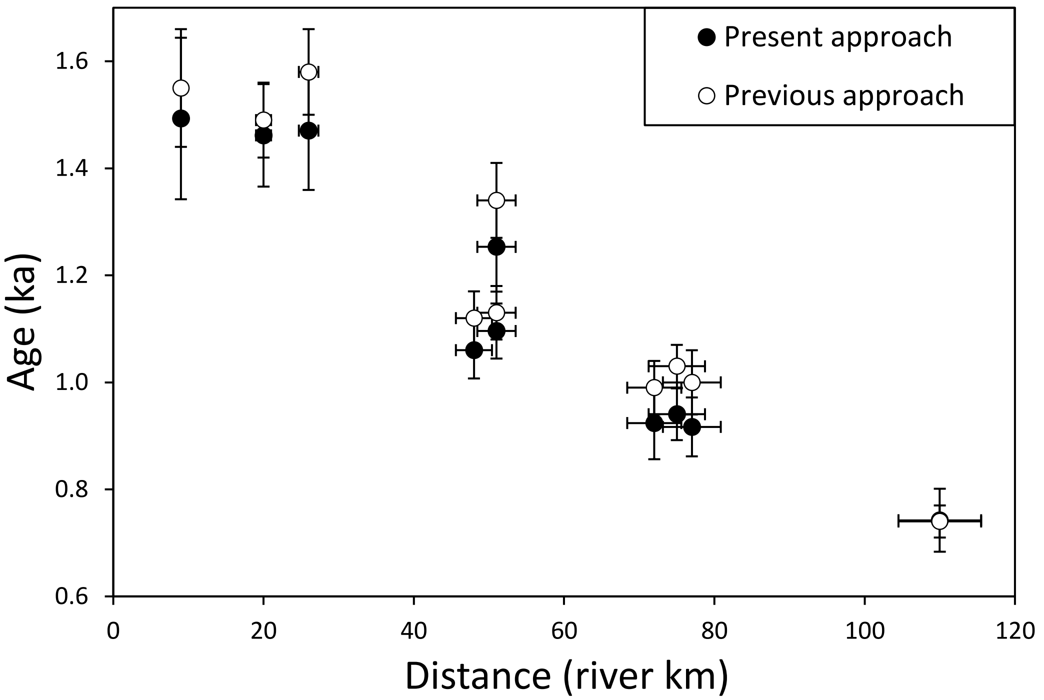

fig. S3. Comparison of mouth bar sand ages estimated using two approaches. Ages obtained with the present OSL dating approach and ages obtained using previously published methods are given. Weighted mean ages with their unshared uncertainties for each cross section are shown versus distance from the polyfurcation point. The ages at Fourchon (river km 110) are nearly identical, although the uncertainty is higher with the present approach.

table S7. Comparison of OSL ages estimated with two approaches. The total uncertainty (arising from shared plus unshared sources) is also provided. Red numbers indicate rejected ages.

Transect	River Km	Sample name	Present approach		Previous approach		$Age_{Previous}$ - $Age_{Present}$
			Age (ka)	Weighted mean (ka)	Age (ka)	Weighted mean (ka)	Age difference (ka)
St. Charles	9	SC I-1	1.49 ± 0.15	1.49 ± 0.15	1.55 ± 0.11	1.55 ± 0.11	0.06 ± 0.19
		SC I-2[a]	1.12 ± 0.10		1.24 ± 0.08		0.13 ± 0.08
		SC I-2[b]	1.28 ± 0.12		1.14 ± 0.10		-0.14 ± 0.10
Bayou Cane	20	BC I-2	1.51 ± 0.12	1.46 ± 0.10	1.44 ± 0.09	1.49 ± 0.07	-0.07 ± 0.13
		BC I-1	1.38 ± 0.14		1.52 ± 0.09		0.14 ± 0.09
Raceland	26	RL I-1	1.61 ± 0.16	1.47 ± 0.11	1.62 ± 0.11	1.58 ± 0.08	0.01 ± 0.16
		RL I-2	1.32 ± 0.14		1.54 ± 0.11		0.22 ± 0.11
Dulac	48	DL I-2	1.08 ± 0.06	1.06 ± 0.05	1.12 ± 0.06	1.12 ± 0.05	0.04 ± 0.08
		DL I-1	1.04 ± 0.07		1.11 ± 0.06		0.07 ± 0.06
Larose	51	LR I-1	1.29 ± 0.09	1.25 ± 0.08	1.38 ± 0.08	1.34 ± 0.07	0.10 ± 0.12
		LR I-2	1.16 ± 0.14		1.29 ± 0.09		0.13 ± 0.09
Chauvin	51	CV I-1	1.08 ± 0.06	1.10 ± 0.05	1.11 ± 0.06	1.13 ± 0.05	0.03 ± 0.08
		CV II-1	1.11 ± 0.06		1.14 ± 0.06		0.03 ± 0.06
Galliano	72	GL I-2	0.89 ± 0.07	0.92 ± 0.07	0.96 ± 0.07	0.99 ± 0.05	0.07 ± 0.10
		GL I-1	1.01 ± 0.14		1.02 ± 0.07		0.01 ± 0.07
Cocodrie	75	CD I-2	0.95 ± 0.06	0.94 ± 0.05	1.05 ± 0.06	1.03 ± 0.04	0.10 ± 0.08
		CD I-1	0.92 ± 0.08		1.02 ± 0.05		0.10 ± 0.05
Golden Meadow	77	GM I-1	0.96 ± 0.06	0.92 ± 0.06	1.07 ± 0.06	1.00 ± 0.06	0.11 ± 0.08
		GM I-2	0.82 ± 0.08		0.93 ± 0.06		0.12 ± 0.06
Fourchon	110	FC I-2	0.77 ± 0.07	0.74 ± 0.06	0.75 ± 0.04	0.74 ± 0.03	-0.02 ± 0.07
		FC I-1	0.59 ± 0.12		0.73 ± 0.03		0.14 ± 0.03
[a]75-125 µm, [b]125-180 µm							
